# Supplementary material for: Splice-Junction-Based Mapping of Alternative Isoforms in the Human Proteome
Source: Cell Rep. Author manuscript; Available in PMC 2020 Jan 15. (PMC6961840; doi:10.1016/j.celrep.2019.11.026)
Supplement: 2 [file NIHMS1546469-supplement-2.zip › DF1/PXD006675/AtrialSeptum/AtrialSeptum_10_PDLIM5_ANLSSSTGNVEDSFEGFR.pdf]

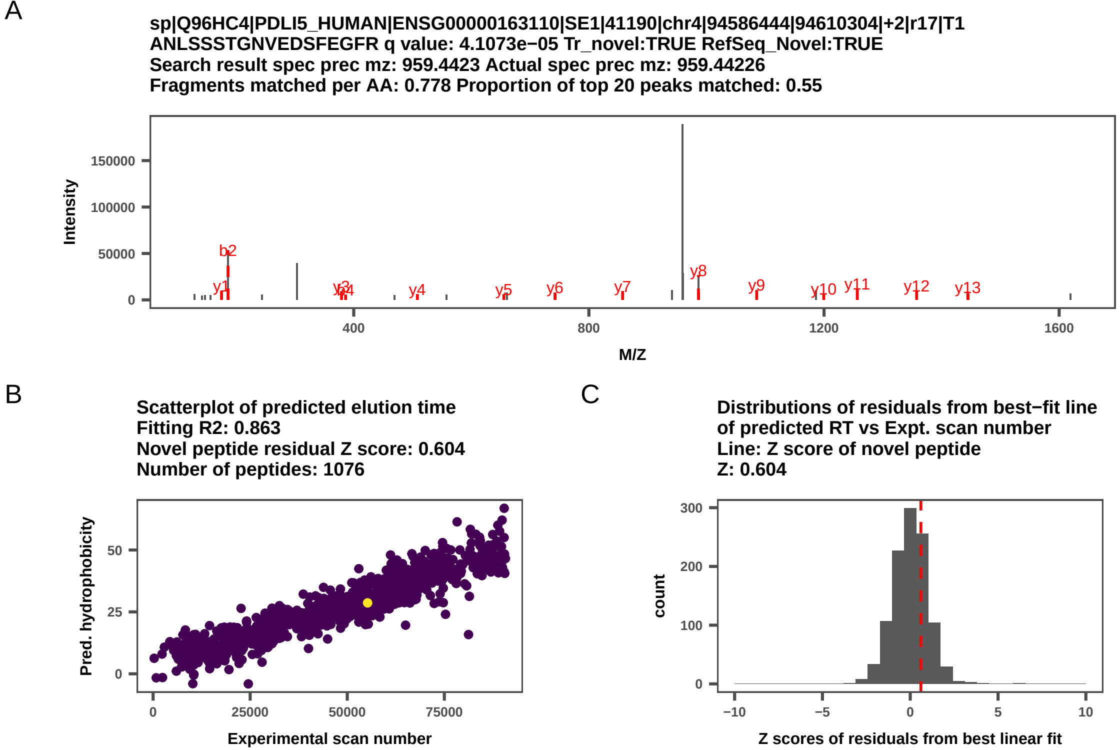
A
sp|Q96HC4|PDLI5_HUMAN|ENSG00000163110|SE1|41190|chr4|94586444|94610304|+2|r17|T1
ANLSSSTGNVEDSFEGFR q value: 4.1073e−05 Tr_novel:TRUE RefSeq_Novel:TRUE
Search result spec prec mz: 959.4423 Actual spec prec mz: 959.44226
Fragments matched per AA: 0.778 Proportion of top 20 peaks matched: 0.55
Intensity
150000
100000
50000
0
b2
y1
y3
b4
y4
y5
y6
y7
y8
y9
y10
y11
y12
y13
400
800
1200
1600
M/Z
B
Scatterplot of predicted elution time
Fitting R2: 0.863
Novel peptide residual Z score: 0.604
Number of peptides: 1076
Pred. hydrophobicity
50
25
0
0
25000
50000
75000
Experimental scan number
C
Distributions of residuals from best−fit line
of predicted RT vs Expt. scan number
Line: Z score of novel peptide
Z: 0.604
count
300
200
100
0
−10
−5
0
5
10
Z scores of residuals from best linear fit
